# Dosimetric feasibility of stereotactic body radiation therapy as an alternative to brachytherapy for definitive treatment of medically inoperable early stage endometrial cancer

**DOI:** 10.1186/1748-717X-9-164

**Published:** 2014-07-24

**Authors:** Ryan Jones, Quan Chen, Ryan Best, Bruce Libby, Edwin F Crandley, Timothy N Showalter

**Affiliations:** 1 Department of Radiation Oncology, University of Virginia School of Medicine, 1215 Lee Street, Box 800383, Charlottesville, VA 22908, USA

**Keywords:** SBRT, Medically inoperable, Endometrial cancer, Intracavitary brachytherapy

## Abstract

**Purpose:**

This study was designed to evaluate the dosimetric feasibility of definitive stereotactic body radiation therapy (SBRT) for the treatment of medically inoperable early stage endometrial cancer.

**Methods:**

CT simulation scans from 10 medically inoperable early stage endometrial cancer patients previously treated with high dose-rate (HDR) intracavitary brachytherapy were used to generate Helical Tomotherapy (HT) plans using the IMRT mode with clinical target volumes (CTVs) that included the uterus plus cervix. A prescription dose of 34 Gy in 4 fractions was used. The SBRT dosimetry was compared to the 10 prior intracavitary brachytherapy plans normalized to a standard dose. Organs at risk (OARs) evaluated were the bladder, rectum, sigmoid, femoral heads, and other bowel, including both large and small bowel. The simulation CT and daily image guidance for 4 patients treated with this technique were evaluated to assess for interfraction variation in the uterine position and effects on dosimetry.

**Results:**

Compared to intracavitary brachytherapy, HT SBRT produced significantly greater overall target coverage to the uterus, boost CTV, and PTV, with exception of the V150% of the uterus. HT SBRT significantly increased dose to the rectum, bowel, and femoral heads compared to intracavitary brachytherapy, though not outside of dose tolerance limits. Review of daily image guidance for patients treated with this technique demonstrated good reproducibility with a mean overlap index of 0.87 (range, 0.74 – 0.99).

**Conclusions:**

Definitive SBRT for medically inoperable early stage endometrial cancer appears to be a feasible treatment option. Future studies are warranted to evaluate long-term clinical outcomes with this technique, compared to HDR intracavitary brachytherapy.

## Background

The American Cancer Society estimates that 49,560 new cases of endometrial cancer will be diagnosed in the United States in 2013 [1]. Nearly 70% of all endometrial cancers in the United States are early stage at disease presentation [2], equating to a predicted 33,701 diagnoses of early stage endometrial cancer in the United States in 2013. Optimally, early stage endometrial cancer is staged and treated with surgery, including a hysterectomy and bilateral salpingo-oophorectomy, with or without lymphadenectomy, and then appropriate adjuvant therapy may be considered [3]. Unfortunately, some patients are ineligible for surgery because of medical conditions such as morbid obesity or cardiopulmonary dysfunction that make surgical or anesthesia too risky [4].

Medically inoperable early stage endometrial cancer is routinely treated with intracavitary brachytherapy using a device such as a Y-applicator or Heyman’s capsules, either alone or in combination with pelvic external beam radiation therapy [5]. Placement of the intracavitary applicators may require the use of general or regional anesthesia in an operating room or dedicated brachytherapy space. The medical contraindications to surgery, such as morbid obesity, advanced age and cardiopulmonary disease, may similarly increase the risks from anesthesia and brachytherapy [6-8]. Published reports suggest that brachytherapy can be delivered safely and effectively for medically inoperable endometrial cancer [4,9], however this patient population is considered at high risk from procedures and anesthesia by virtue of classification as medically inoperable. Nguyen and Petereit reported outcomes of 36 women with medically inoperable endometrial cancer who received high-dose-rate (HDR) brachytherapy at a single institution; they observed a 21% rate of late complications and 2 patients died from acute cardiovascular events within 30 days of treatment [10]. At our institution, due to the invasiveness of the procedure and/or risks of anesthesia, it is not uncommon for these patients or their families to refuse brachytherapy or for patients to be considered ineligible for anesthesia.

An external beam radiation therapy (EBRT)-only strategy for the definitive treatment of early stage medically inoperable endometrial cancer could offer some advantages for these patients since EBRT would avoid the risk of anesthesia, applicator placement and immobilization. However, EBRT has some general limitations for such treatment and the physical characteristics of EBRT are inherently inferior to brachytherapy. Without brachytherapy, EBRT alone suffers from inability to generate comparatively high doses to tumors otherwise accessible by brachytherapy, thus there is a theoretical risk of decreased local tumor control. Additionally, studies of interfraction uterine positioning during definitive RT for cervical cancer [11,12] raise concerns regarding day-to-day motion that theoretically may further complicate the local tumor control and normal tissue toxicity from EBRT for medically inoperable endometrial cancer. Brachytherapy avoids concern, since it is placed directly within the uterus target volume.

For patients at highest risk of complications from anesthesia and brachytherapy, stereotactic body radiation therapy (SBRT) may allow for the reproducible delivery of high doses of radiation to the uterus and providing a back-up alternative for select patients. Kemmerer et al. explored the utility of EBRT with an SBRT boost for medically inoperable endometrial cancer and found the option to be safe and effective for the early stage patients in their cohort [13]. In the current study, we further develop the concept of SBRT for medically inoperable early stage endometrial cancer with the aim of providing a potential alternative to brachytherapy when it is considered medically necessary to avoid the risks of anesthesia, immobilization, and applicator placement.

Here, we report a dosimetric evaluative study that compares a proposed SBRT approach using Helical Tomotherapy (Accuray Inc., Sunnyvale, CA) to high-dose-rate (HDR) intracavitary brachytherapy for medically inoperable, (FIGO) Stage I-II endometrial cancer patients. In addition, we identify appropriate dose-volume parameters for the SBRT strategy and evaluate the interfraction motion observed among 4 consecutive patients treated at our institution who were treated with EBRT.

## Methods

### Subjects

With institutional review board approval, we identified 10 patients who received HDR intracavitary brachytherapy at our institution for medically inoperable, FIGO Stage I-II endometrial cancer in order to perform a dosimetric analysis of SBRT versus HDR brachytherapy. Among these 10 subjects, the contributing comorbidities to medically inoperable status included: morbid obesity (n = 6), cardiovascular dysfunction (n = 6), severe anemia (n = 1) and end-stage cirrhosis (n = 1). A Y-shaped applicator (two tandems) was used for 3 patients, while a single tandem was used for 7 patients. The Y-applicators were placed under general anesthesia, and the single tandems were placed under mild sedation. There were no cases where Heyman’s capsules were used, since this approach is not used at our institution. Five patients received HDR brachytherapy alone, and 5 patients received brachytherapy combined with pelvic EBRT. Perioperative complications included severe pain (n = 3), uterine perforation (n = 1), and decreased oxygen saturation (n = 2). Interfraction variation in uterine position was studied using treatment planning and daily image guidance scans for a separate group of 4 patients who received EBRT for medically inoperable uterine cancer at our institution.

### Treatment planning

In order to permit dosimetric comparison of the HDR intracavitary brachytherapy to the proposed SBRT approach, the brachytherapy plans for each patient were copied and rescaled in the BrachyVision® (Varian Medical Systems, Palo Alto, CA) treatment planning system (TPS) to reflect a standardized prescription of 34 Gy in 4 fractions to the clinical target volume (CTV), defined as the uterus plus the cervix. This dose is consistent with American Brachytherapy Society guidelines for HDR brachytherapy monotherapy for inoperable endometrial cancer [5]. The CT scan from brachytherapy included the applicator as placed by the physician for brachytherapy, with the intent of representing actual, rather than ideal, coverage. The target volume coverage by the prescription dose is anticipated to vary with quality of applicator placement, and to be lower for single tandem than for Y-applicator plans. Treatment planning involved three-dimensional planning, which has been shown to reduce doses to normal tissue structures in this setting [14]. All CT images were obtained on a standard CT simulator, and the scans were exported with 2–3 mm slice thickness. The remaining contoured volumes included the following organs-at-risk (OARs): bladder, rectum, sigmoid colon, other bowel (all individual loops of large and small bowel within 1 jaw width of the uterus), and bilateral femoral heads.

The same 10 CT scans and all contours were transferred from the BrachyVision® TPS to the Tomotherapy TPS. SBRT planning was conducted on the Tomotherapy TPS using the following parameters: intensity modulated radiation therapy (IMRT) mode, 2.5 cm jaw width, pitch of 0.287, and modulation factor of 2.5. The CTV was again defined as the uterus plus the cervix and was set as the prescription structure, while a 1 cm uniform volumetric contraction of the uterus was used to generate a boost CTV. The 1 cm contraction was chosen empirically as an approximation of the endometrial surface. A planning target volume (PTV) was generated based upon a 2 mm symmetric expansion of the primary CTV (uterus plus cervix). Target dose prescriptions for the SBRT treatments included the following: a minimum of 90% of the CTV to receive 34 Gy in 4 fractions (equivalent dose in 2-Gy fractions (EQD_2_) of 52.4 Gy), a minimum of 90% of a Boost CTV to receive 48 Gy in 4 fractions (EQD_2_ of 88.0 Gy), and a minimum of 90% of an expansion PTV to receive 21 Gy in 4 fractions (EQD_2_ of 26.7 Gy). The dose fractionation for SBRT and use of a Boost CTV structure was chosen to simulate standard HDR brachytherapy dose distributions delivered as monotherapy for medically inoperable endometrial cancer [5].

### Dosimetric endpoints evaluated

As mentioned, the following OARs were contoured for both brachytherapy and SBRT plans: bladder, rectum, sigmoid colon, other bowel (all individual loops of large and small bowel within 1 jaw width of the uterus), and bilateral femoral heads. Contouring and dose restriction of the bowel was necessary to yield bowel doses inside of tolerance limits for the SBRT plans. Normal tissue dose tolerance limits were set using recommendations from the American Association of Physicists in Medicine (AAPM) Group 101, a pelvic SBRT RTOG trial, (0938) and guidelines from the American Brachytherapy Society (ABS) and the gynecological working group of GEC-ESTRO (Groupe Européen de Curiethérapie and European Society for Radiotherapy & Oncology) (see Table 1) [15-18]. When needed, the biological effective dose (BED) of these limits were calculated and converted to the 4-fraction treatment equivalent. BED was calculated by

$$\mathrm{BED}\;=\;\mathrm{nd}\left(1+\frac{d}{\alpha /\beta }\right)$$

where *d* is the fractional dose and *n* is the number of fractions. The limit BEDs were calculated using an alpha-beta ratio (α/β) of 10, traditionally viewed as the α/β of acute effects. In addition to those data points, traditionally important parameters in brachytherapy and SBRT were also included. The final list of examined dosimetric parameters included: highest dose encompassing 90% (D90%) of the Boost CTV; D90% of PTV; uterus volume (uterus plus cervix) D95%, D90%, D50%, and volume receiving 150% of the prescription dose (V150%); the dose received by the hottest 0.1 cc (D0.1 cc), 1 cc (D1 cc), and 2 cc (D2 cc) for the bladder, rectum and sigmoid, and additional dose-volume points shown in Table 2. Dosimetric statistics for the brachytherapy plans were extracted directly from the BrachyVision® software. The dose-volume histograms for the SBRT plans were first converted into text file (.txt) format and then analyzed using 2011 Excel software (Microsoft, Redmond, WA) in order to obtain the data point values. After confirming that the data were normal distributed, statistical comparisons between SBRT and brachytherapy plans were performed using two-tailed T test with threshold for significance of *p* < 0.05.

**Table 1:** Normal tissue dose tolerance limits for 4-fraction stereotactic body radiation therapy

**Site**	**4-fraction SBRT**		**Data point**	**Reference**	**Reference limit**	**Reference BED (α/β = 10)**	**Current BED (α/β = 10)**
	**Volume**	**Threshold dose (Gy) per fx**					
Bladder							
	<1 cc	8.93	1 cc	RTOG 0938	<105% Rx	67	67.6
	<2 cc	4.60	2 cc	Institutional*	4.8 Gy × 4	--	--
	<15 cc	4.50	15 cc	TG-101	5.6 Gy × 3	26.2	26.1
	90%	7.65	D10%	RTOG 0938	<90% Rx	53.9	54.0
	50%	4.25	D50%	RTOG 0938	<50% Rx	24.7	24.2
Rectum							
	<1 cc	8.93	1 cc	RTOG 0938	<105% Rx	67	67.6
	<2 cc	4.60	2 cc	Institutional*	4.8 Gy × 4	--	--
	<3 cc	8.08	3 cc	RTOG 0938	<95% Rx	58.1	58.4
	<20 cc	6.50	20 cc	TG-101	8 Gy × 3	43.2	42.9
	90%	7.65	D10%	RTOG 0938	<90% Rx	53.9	54.0
	80%	6.80	D20%	RTOG 0938	<80% Rx	45.8	45.7
	50%	4.25	D50%	RTOG 0938	<50% Rx	24.7	24.2
Sigmoid colon							
	<2 cc	4.80	2 cc	Institutional*	4.8 Gy × 4	--	--
	<20 cc	6.50	20 cc	TG-101	8 Gy × 3	43.2	42.9
Other bowel							
Small bowel	<5 cc	4.75	5 cc	TG-101	5.9 Gy × 3	28.1	28.0
Large bowel	<20 cc	6.50	20 cc	TG-101	8 Gy × 3	43.2	42.9
Femoral Heads							
	Point	6.89	Max	RTOG 0938	<81% Rx	46.6	46.5
	<10 cc	4.59	10 cc	RTOG 0938	<54% Rx	27.2	26.8

These are proposed guidelines that were used as constraints in the current study. References for each parameter are listed.

*In accordance with our institutional per-fraction brachytherapy policy.

*Abbreviations: SBRT* stereotactic body radiation therapy, *fx* fraction, *BED* biological effective dose, *Rx* prescription dose, *D(x)%* dose to x percent of the volume, *RTOG* Radiation Therapy Oncology Group, *TG-101* stereotactic body radiation therapy Task Group 101.

**Table 2:** Normal tissue dosimetric comparison of stereotactic body radiation therapy and intracavitary brachytherapy

**Site**	**Volume**	**Tolerance limit**	**Mean dose (Gy, per fx) ± SD**		***p*-value**
			**I-BT**	**SBRT**	
*Bladder*					
	D50%	4.25	2.04 ± 0.78	1.76 ± 1.08	NS
	D10%	7.65	3.73 ± 1.44	3.65 ± 1.37	NS
	D0.1 cc	-	6.44 ± 3.05	5.55 ± 1.46	NS
	D1 cc	8.93	5.22 ± 2.27	4.76 ± 1.48	NS
	D2 cc	4.60	4.68 ± 2.02	4.34 ± 1.51	NS
*Rectum*					
	D50%	4.25	0.83 ± 0.37	1.20 ± 0.63	NS
	D20%	6.80	1.27 ± 0.85	2.25 ± 0.76	NS
	D10%	7.65	1.56 ± 1.21	2.65 ± 0.75	NS
	D0.1 cc	-	2.64 ± 2.68	4.25 ± 1.25	NS
	D1 cc	8.93	2.14 ± 1.98	3.37 ± 0.80	NS
	D2 cc	4.60	1.91 ± 1.65	3.07 ± 0.74	NS
	D20 cc	6.50	0.93 ± 0.50	1.40 ± 0.84	NS
*Sigmoid*					
	D0.1 cc	-	5.41 ± 1.66	5.77 ± 0.52	NS
	D2 cc	4.80	4.05 ± 1.18	4.48 ± 0.49	NS
	D20 cc	6.50	1.73 ± 0.82	2.65 ± 0.78	0.02
*Other bowel*					
	D0.1 cc	-	3.49 ± 1.43	6.69 ± 0.55	<0.005
	D2 cc	-	2.66 ± 1.11	5.56 ± 0.45	<0.005
	D5 cc	4.75	2.28 ± 0.99	5.03 ± 0.46	<0.005
	D20 cc	6.50	1.51 ± 0.64	3.94 ± 0.62	<0.005
*Femoral Heads*					
	Max	6.89	0.83 ± 0.22	2.83 ± 1.22	<0.005
	D10 cc	4.59	0.57 ± 0.15	2.22 ± 1.09	<0.005

*Abbreviations: SD* standard deviation, *I-BT* intracavitary brachytherapy, *SBRT* stereotactic body radiation therapy, *fx* fraction, *V(x)%* the percentage volume of the structure receiving *x* percent of the prescription dose of 8.5Gy, *D(x)%* the highest dose received by *x* percent of the volume, *NS* non-significant.

### Interfraction variation in uterine position

Evaluation of interfraction variation of the uterus for 4 consecutive medically inoperable endometrial cancer patients treated with EBRT alone at our institution was performed by comparing uterine position on the simulation CT to daily imaging from the treatment machine. Patients were simulated in the supine position and were immobilized using the BodyFix system. Patients were instructed to empty their bladder prior to simulation and prior to each fraction of radiation therapy. No specific bowel preparatory instructions were given. Prior to each treatment on the Tomotherapy HD unit, an MVCT was obtained which included the entire uterus and shifts were made to optimize soft tissue alignment. Each pre-treatment MVCT image was co-registered to the respective treatment planning CT in Velocity (Velocity Medical Solutions, Atlanta, GA) using the shifts applied on the treatment machine. The CTV uterus was contoured on each MVCT for comparison to the simulation CT. Two metrics were used assess for interfraction reproducibility of the uterine position: (1) overlap index and (2) daily D90 of the CTV uterus. The overlap index was defined as: (volume of CTV uterus on the MVCT overlapping with the CTV uterus from the treatment planning CT)/(volume of CTV uterus from the treatment planning CT). The daily D90 for the CTV uterus was obtained from dose volume histogram data in Velocity after co-registration of the daily MVCT with the treatment planning CT.

## Results

**Figure 1:**
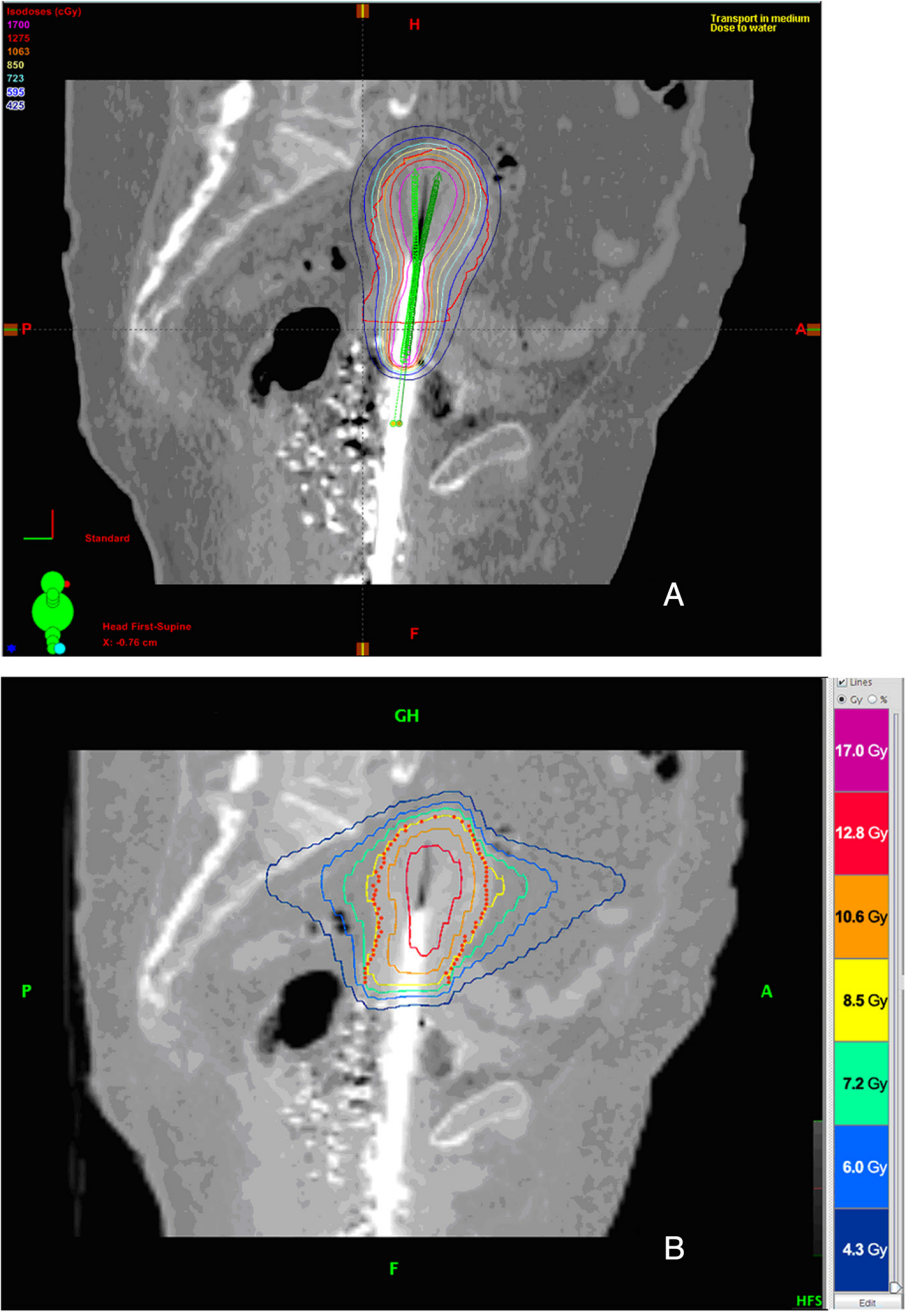
**Sagittal images from treatment plans for HDR brachytherapy (A) and Tomotherapy SBRT (B) approaches for a single patient with medically inoperable endometrial cancer.** Both treatment plans were performed with goal of delivering 8.5 Gy per fraction to the uterus.

We offer the computations in Table 1 as future reference for normal dose limits for 4-fraction pelvic SBRT treatments. In this study, HT SBRT achieved most of the pre-specified normal tissue dose-volume constraints for all ten patients evaluated (Table 2). However, the D5 cc goal for small bowel was exceeded in Tomotherapy plans for 8 of 10 patients. The target dose limit for D5 cc of small bowel was 4.75 Gy per fraction, and the 10 HT SBRT plans yielded an average D5 cc of 5.03 ± 0.46 Gy. There were 6 patients whose HDR brachytherapy plans did not meet specified bladder 2 cc goals. For 4 of these 6 cases, the SBRT plans also did not meet bladder 2 cc goals; however, SBRT plans achieved bladder goals in 2 of these 6 patients. The overall average bladder 2 cc goals were met for both HDR brachytherapy and SBRT. Rectum 2 cc constraints were exceeded in 1 HDR brachytherapy plan, and the SBRT plan for that patient was able to meet that goal. Sigmoid 2 cc constraints were exceeded in 5 HDR brachytherapy plans and 3 SBRT plans. The overall average sigmoid 2 cc goals were met for both HDR brachytherapy and SBRT. A representative comparison of HDR brachytherapy and SBRT plans is shown in Figure 1, which depicts a mid-sagittal image from the treatment plans for a single patient evaluated in this study.

The average and standard deviation values for each dose-volume parameter for HDR brachytherapy plans and Tomotherapy SBRT plans are displayed in Tables 2 and 3, along with *p* value for statistical comparisons between SBRT and brachytherapy plans. In general, SBRT treatment planning achieved higher percentage of target volume coverage than brachytherapy (Table 3). For example, the average D95% for the uterus was over 350% higher for SBRT compared to brachytherapy (8.34 ± 0.05 Gy per fraction versus 2.34 ± 1.26 Gy, respectively; *p* < 0.001). However, brachytherapy plans were associated with higher average V150% for the uterus (22.7%) than SBRT plans (16.7%) (*p* = 0.025).

**Table 3:** Target volume dosimetric comparison of stereotactic body radiation therapy and intracavitary brachytherapy

**Site**	**Volume**	**Mean dose (per fx) ± SD**		***p-value***
		**I-BT**	**SBRT**	
*Uterus*				
	V150%	22.66 ± 7.21	16.66 ± 5.44	0.05
	D95% (Gy)	2.34 ± 1.26	8.34 ± 0.05	<0.005
	D90% (Gy)	2.76 ± 1.44	8.69 ± 0.09	<0.005
	D50% (Gy)	6.06 ± 2.88	10.48 ± 0.31	<0.005
*BoostCTV*				
	D90% (Gy)	5.62 ± 3.79	12.10 ± 0.45	<0.005
*PTV*				
	D90% (Gy)	2.48 ± 1.27	7.98 ± 0.25	<0.005

*Abbreviations: SD* standard deviation, *I-BT* intracavitary brachytherapy, *SBRT* stereotactic body radiation therapy, *fx* fraction, *V150%* the percentage volume of the structure receiving *150* percent of the prescription dose of 8.5 Gy, *D(x)%* the highest dose received by *x* percent of the volume.

### Interfraction variation in uterine position

Pre-treatment MVCT imaging was reviewed for 4 patients, for 4 fractions each, for a total of 16 MVCT scans. For the entire cohort, the mean overlap index was 0.87 (range, 0.74 – 0.99). For the entire cohort, the mean D90 for coverage of CTV uterus was 99.4% of the prescription dose (range, 84.2 – 110.0%). Data are summarized in Table 4.

**Table 4:** Summary of calculated D90 (as percentage of prescription dose) and overlap index for 4 patients who received external beam radiation therapy for medically inoperable endometrial cancer

**Patient 1**	**D90 (% prescription dose)**	**Overlap index**
CTV uterus	110.2	
Fraction 1	110.0	0.86
Fraction 2	97.4	0.74
Fraction 3	105.7	0.89
Fraction 4	101.1	0.83
**Patient 2**	**D90 (% prescription dose)**	**Overlap index**
CTV uterus	100.7	
Fraction 1	100.6	0.92
Fraction 2	100.6	0.93
Fraction 3	100.5	0.89
Fraction 4	96.4	0.80
**Patient 3**	**D90 (% prescription dose)**	**Overlap index**
CTV uterus	100.5	
Fraction 1	96.9	0.83
Fraction 2	99.8	0.89
Fraction 3	100.3	0.92
Fraction 4	100.4	0.92
**Patient 4**	**D90 (% prescription dose)**	**Overlap index**
CTV uterus	103.9	
Fraction 1	87.4	0.86
Fraction 2	98.8	0.90
Fraction 3	84.2	0.82
Fraction 4	109.7	0.99
**Mean**	99.4	0.87
**Range**	84.2 - 110.0	0.74 - 0.99

The values reflect calculation of radiation doses on megavoltage CT image guidance scans obtained at the time of treatment, using sample SBRT plans.

## Discussion

In this study, we compared dosimetric parameters observed with Helical Tomotherapy SBRT to those of intracavitary brachytherapy for the definitive treatment of medically inoperable early stage endometrial cancer. In all 10 cases, the SBRT achieved higher percentage overall dosimetric coverage of the uterus, Boost CTV, and PTV, as demonstrated by the D90%. However, intracavitary brachytherapy plans yielded a greater volume of the uterus receiving 150% of the prescription dose, which suggests that brachytherapy would achieve higher likelihood of tumor control at a given prescription dose. Since the brachytherapy cases included the some single-tandem applications, which result in inferior coverage compared to Y-applicators, the observed coverage difference between SBRT and brachytherapy may be overestimated in this study. The SBRT plans achieved most normal tissue dose constraints, but HT SBRT was associated with increased doses to the sigmoid colon, bowel and femoral heads compared to brachytherapy. Given the lack of data on clinical outcomes, the reduced V150% for the CTV and the higher doses to the bowel suggest that SBRT warrants further study before being implemented in the clinic. Review of daily image guidance scans for a cohort of 4 patients who received EBRT for medically inoperable endometrial cancer at our institution suggests that there is good reproducibility of uterine target volume positioning and dosimetry between fractions. However, the number of patients evaluated in this study is small, and concerns remain regarding interfraction uterine motion. Based on observations in the current study, SBRT is a reasonable potential back-up alternative to intracavitary brachytherapy for those medically inoperable endometrial cancer patients who are at high risk of procedural or anesthesia-related complications, but this study also highlights the superiority of brachytherapy with respect to delivering very high doses to tumors (e.g., V150%) and reducing dose to OARs (e.g., bowel).

Although the current study evaluates dosimetric characteristics of HT SBRT plans, relative to brachytherapy, conclusions are limited by the lack of evidence regarding tumor control and toxicity outcomes with this treatment. In this study, definitive SBRT produced increased overall dose to the target volume compared to brachytherapy, while lowering the V150% of the target. Theoretically, the significant volume of tumor exposed to higher doses during brachytherapy treatment may be of greater importance for tumor control than percentage coverage by the prescription dose, so data regarding clinical outcomes after SBRT are needed before adopting this approach widely. Our SBRT approach attempts to address this issue, in part, by including a target volume that receives 48 Gy. This consideration must be balanced against the potential advantage of avoiding the risks of anesthesia and the brachytherapy procedure itself for patients with severe medical comorbidities.

There are only a limited number of series that report outcomes after definitive brachytherapy for medically inoperable endometrial cancer, but the outcomes are generally good. Coon et al. utilized either a combination of EBRT and Y-applicator brachytherapy (71%) or Y-applicator brachytherapy alone (29%) for the treatment of medically inoperable early stage endometrial cancer and reported 3- and 5-year actuarial cause-specific survival rates of 93% and 87%, respectively [4]. Niazi et al. utilized either a combination of EBRT and Y-applicator brachytherapy (21%) or Y-applicator brachytherapy alone (79%) and found an average 15-year disease-specific survival rate of 78% for all stages [9]. Prospective studies of SBRT for medically inoperable endometrial cancer are warranted to evaluate outcomes relative to these benchmark data from brachytherapy alone and in combination with conventionally fractionated EBRT.

Our analysis indicates that acceptable radiation therapy plans are achievable using this technique, however, this treatment technique results in high dose distributions with steep gradients targeting a mobile organ. While interfraction and intrafraction motion is also a consideration in SBRT for other disease sites, the uterus is unique in that there is potential for deformation of the target shape due to variations in bladder and rectal filling. Several studies have demonstrated significant interfraction motion of the uterus during definitive EBRT for gynecological tumors [11,12], raising concerns for the ability to safely deliver SBRT. However, it is not clear that data regarding uterine interfraction motion in cervical cancer patients, as included in those studies, can be applied to patients with medically inoperable endometrial cancer, who are often elderly and may have smaller uteri. Our data indicate that after daily image guidance with soft tissue alignment, excellent reproducibility in uterine positioning is achievable. Moreover, when the isodose distributions from the treatment planning CTs were used to estimate daily dose delivery to the uterus, the mean D90 for the cohort was 99.4% of the prescription dose. This indicates that despite small residual errors in uterine positioning, treatment delivery with this technique results in excellent coverage of the target volume. An additional consideration for uterine SBRT is the additional exposure to low-dose irradiation, which has the theoretical potential to increase the risk of secondary malignancies. Given the limited life expectancy of many patients with medically inoperable endometrial cancer, this is likely not a primary factor for decision-making.

The current study has limitations, most notably the lack of data regarding clinical outcomes. Prospective clinical trials of SBRT are needed to develop this approach for implementation. In addition, the brachytherapy treatment plans used as the comparison in this study include a majority of cases with single tandem applicator rather than a Y applicator. Therefore, the CTV coverage provided by brachytherapy in the current study likely underestimates what would be achieved with a Y applicator, which is a preferred treatment approach over single tandem brachytherapy [4,5]. The current study was limited to analysis of brachytherapy alone versus SBRT alone, and did not address the scenario of pelvic nodal EBRT combined with brachytherapy or SBRT. It is possible that 2 fractions of SBRT (8.5 Gy × 2) could be explored as a potential alternative to brachytherapy when pelvic EBRT will also be delivered. This would mimic the addition of 2 fractions of HDR brachytherapy (8.5 Gy × 2) to 45 Gy of pelvic EBRT, which is one of the recommended dose schedules in ABS guidelines [5]. Furthermore, it should be noted that interfraction variation of the uterus was evaluated in a small number of patients in this study, and additional research is warranted to assess the significance of organ motion for patients with medically inoperable endometrial cancer. We do not recommend that SBRT be pursued for all patients with medically inoperable endometrial cancer, since HDR brachytherapy is generally considered to be safe and effective [4,9], but rather only in those individuals who are considered to be at unacceptably high risk of complications from anesthesia, such as the group described by Kemmerer and colleagues in their evaluation of SBRT as a boost strategy in lieu of brachytherapy [13]. We would recommend rigid immobilization for uterine SBRT, as well as consideration of repeat volumetric imaging during SBRT if the treatment delivery time is expected to be more than a few minutes.

Many brachytherapists may be reluctant to contemplate SBRT as a potential back-up alternative to brachytherapy for any indication, but it is relevant to consider that patterns of cares studies have shown lower than expected rates of utilization of brachytherapy for gynecological cancers [19], reduced utilization of brachytherapy for elderly women with cervical cancer [20], and a recent trend towards decreasing utilization of brachytherapy for cervical cancer in recent years [21]. Therefore, there may be value in better exploring and evaluating EBRT-based approaches as an alternative to brachytherapy based upon the practical extrapolation of these patterns of care studies, which would suggest that some patients with medically inoperable endometrial cancer are likely to receive treatment without brachytherapy despite the lack of evidence.

## Conclusions

In summary, the SBRT technique evaluated in this study for medically inoperable uterine cancer seems feasible with respect to achieving dosimetric objectives, however concerns remain regarding the effectiveness of SBRT relative to brachytherapy. With daily image guidance using soft tissue alignment, adequate coverage of the uterus was achieved, but this early observation should be evaluated in a larger cohort. This analysis demonstrated significant limitations of SBRT, including higher doses to bowel and rectum than with brachytherapy as well as interfraction variation in target volume coverage. This treatment approach appears to be a reasonable potential alternative to intracavitary HDR brachytherapy for patients with medically inoperable endometrial cancer at highest risk of complications from anesthesia and brachytherapy, but future studies will be needed to evaluate clinical outcomes after SBRT and the comparative effectiveness relative to brachytherapy.

## Abbreviations

SBRT: Stereotactic body radiation therapy; EBRT: External beam radiation therapy; FIGO: the International Federation of Gynecology and Obstetrics; HDR: High dose rate; HT: Helical tomotherapy; CTV: Clinical target volume; PTV: Planning target volume; HT: Helical tomotherapy; IMRT: Intensity modulated radiation therapy; OAR: Organ at risk; TPS: Treatment planning system; EQD_2_: Equivalent dose in 2 Gy fractions; AAPM: American association of physicists in medicine; RTOG: Radiation Therapy Oncology Group; ABS: American brachytherapy society; GEC-ESTRO: Groupe Europeen de Curietherapie and European society for radiotherapy & oncology; BED: Biological effective dose; CT: Computed tomography; MVCT: Megavoltage CT; Gy: Gray; D90: Highest dose covering 90% of the target volume; V100: Volume encompassed by 100% of the prescription dose.

## Competing interests

The authors declare that they have no competing interests.

## Authors’ contributions

All authors contributed to the data collection and analysis. QC and RJ performed the external beam radiation therapy treatment planning. BL and RB performed the brachytherapy treatment planning. RJ and TNS performed the statistical analysis. All authors contributed to drafting of the manuscript. All authors read and approved the final manuscript.
